# Assessing Usability and Ambulatory Clinical Staff Satisfaction with Two Electronic Health Records

**DOI:** 10.1055/a-2074-1665

**Published:** 2023-06-28

**Authors:** Brian Lefchak, Susan Bostwick, Sarah Rossetti, Kenneth Shen, Jessica Ancker, Kenrick Cato, Erika L. Abramson, Charlene Thomas, Linda Gerber, Amanda Moy, Mohit Sharma, Jonathan Elias

**Affiliations:** 1 NewYork-Presbyterian Hospital, New York, New York, United States; 2 Department of Pediatrics, Weill Cornell Medical Center, New York, New York, United States; 3 Department of Biomedical Informatics, Columbia University, New York, New York, United States; 4 Columbia University School of Nursing, New York, New York, United States; 5 Department of Population Health Sciences, Weill Cornell Medicine, New York, New York, United States

**Keywords:** electronic health records, user-centered design, medical informatics applications, job satisfaction

## Abstract

**Background** A growing body of literature has linked usability limitations within electronic health records (EHRs) to adverse outcomes which may in turn affect EHR system transitions. NewYork-Presbyterian Hospital, Columbia University College of Physicians and Surgeons (CU), and Weill Cornell Medical College (WC) are a tripartite organization with large academic medical centers that initiated a phased transition of their EHRs to one system, EpicCare.

**Objectives** This article characterizes usability perceptions stratified by provider roles by surveying WC ambulatory clinical staff already utilizing EpicCare and CU ambulatory clinical staff utilizing iterations of Allscripts before the implementation of EpicCare campus-wide.

**Methods** A customized 19-question electronic survey utilizing usability constructs based on the Health Information Technology Usability Evaluation Scale was anonymously administered prior to EHR transition. Responses were recorded with self-reported demographics.

**Results** A total of 1,666 CU and 1,065 WC staff with ambulatory self-identified work setting were chosen. Select demographic statistics between campus staff were generally similar with small differences in patterns of clinical and EHR experience. Results demonstrated significant differences in EHR usability perceptions among ambulatory staff based on role and EHR system. WC staff utilizing EpicCare accounted for more favorable usability metrics than CU across all constructs. Ordering providers (OPs) denoted less usability than non-OPs. The Perceived Usefulness and User Control constructs accounted for the largest differences in usability perceptions. The Cognitive Support and Situational Awareness construct was similarly low for both campuses. Prior EHR experience demonstrated limited associations.

**Conclusion** Usability perceptions can be affected by role and EHR system. OPs consistently denoted less usability overall and were more affected by EHR system than non-OPs. While there was greater perceived usability for EpicCare to perform tasks related to care coordination, documentation, and error prevention, there were persistent shortcomings regarding tab navigation and cognitive burden reduction, which have implications on provider efficiency and wellness.

## Background and Significance

The adoption of the Health Information Technology for Economic and Clinical Health (HITECH) Act of 2009 promoted the widespread adoption of electronic health records (EHRs). 1 While EHRs have been associated with improvements in various clinical and organizational outcomes, a growing body of literature has also linked EHRs to documentation burden, clinical burnout, job dissatisfaction, and patient safety concerns. 1 2 3 According to previous studies, usability may be one characteristic hindering EHR function. 4 EHRs often do not match end-user expectations and inadvertently increase cognitive burden as providers attempt to “balance an increase in tasks with no increases in time allotted.” 5 6

The International Organization for Standardization defines usability as “the extent to which a product can be used by specified users to achieve specified goals with effectiveness, efficiency, and satisfaction in a specified context of use.” 4 Usability has been further characterized by Nielsen as learnability, efficiency, memorability, error limitation, and satisfaction. 7 One validated method of assessing usability is the Health Information Technology Usability Evaluation Scale (Health-ITUES) developed by Yen et al and adapted from the Technology Acceptance Model and IBM Computer System Usability Questionnaire. 8 The Health-ITUES evaluates user engagement through customizable questions based on four usability constructs: Quality of Work Life (QWL), Perceived Usefulness (PU), Perceived Ease of Use (PEU), and User Control (UC). 8 As standard industry usability heuristics, Yen et al defined each as follows: QWL evaluates “system impact beyond the system functionality,” PU assesses “system usefulness for a targeted task,” PEU evaluates “user-system interaction,” and UC reflects “user control ability.” 9 Each construct, in turn, evaluates separate usability concepts with internal consistency reliability. 8

NewYork-Presbyterian (NYP) is a large nonprofit academic medical system in New York City metro area with multiple campuses and is affiliated with Columbia University College of Physicians and Surgeons (CU) and Weill Cornell Medical College (WC) and has nearly 20,000 employees overall and over 2,000 hospital beds. Starting in 2019, NYP, CU, and WC initiated a phased transition of their clinical campus EHRs to one system, EpicCare (Epic Systems, Madison, Wisconsin, United States), thereby decommissioning multiple other EHR systems including various iterations of Allscripts (Allscripts Healthcare Solutions, Chicago, Illinois, United States).

In order to study user perceptions on usability, the Epic Pre and Post-Implementation Study Team, a team of clinicians, academic professors, and students across NYP, CU, and WC, adapted and applied the Health-ITUES with assistance from creator Po-Yin Yen into a 19-question electronic survey utilizing the Health-ITUES constructs QWL, PU, PEU, and UC with an additional two-question inquiry on Cognitive Support and Situational Awareness (CSSA). 4 This work was described in Elias et al and found significant differences in usability perceptions based on roles and settings. 4 Clinical staff with prior EHR experience, those working in multiple settings, or in ordering provider (OP) roles, defined as physicians, physician assistants, and nurse practitioners, consistently denoted less usability. 4 Collectively, these results suggest several hypotheses on the burden of multiple EHR system proficiency for select end users; however, differences in usability perceptions related to specific EHR systems remained less well characterized.

**Fig. 1:**
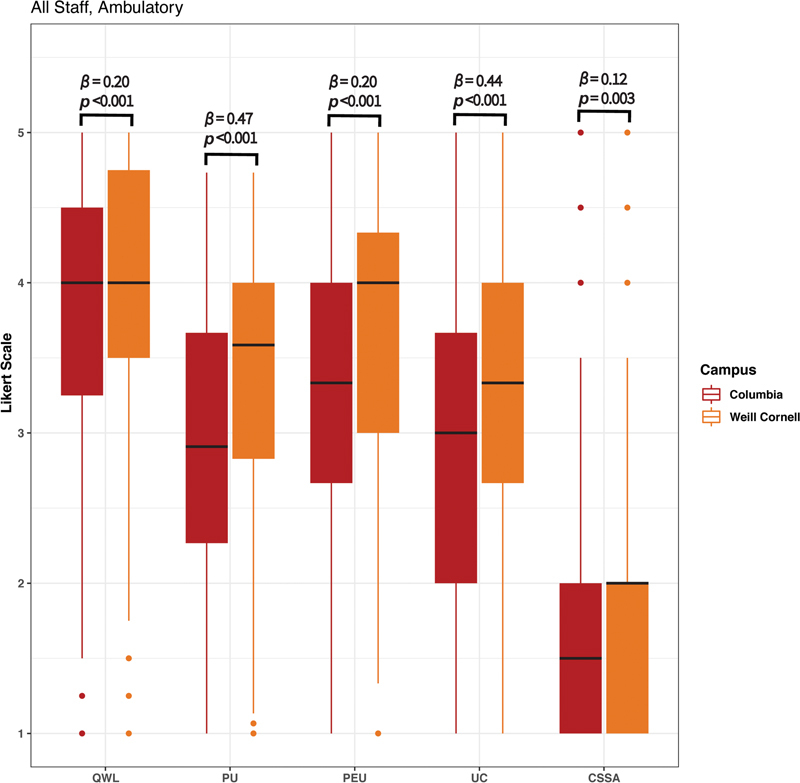
Ambulatory responses of all clinical staff at both Columbia University College of Physicians and Surgeons (CU) and Weill Cornell Medical College (WC).

Given significant differences in EHR usability perceptions based on role, setting, and EHR system, the objective of this study was to further characterize perceptions among clinical staff utilizing EpicCare and various iterations of Allscripts within ambulatory settings of a major academic health care system prior to EHR transition. Understanding differences in usability perceptions may offer critical insight into predictions for future system transitions and occupational satisfaction. 4 7 While previous studies have produced mixed results regarding the impact of EHR transitions on business productivity metrics, fewer studies have assessed metrics specific to users' usability perceptions during EHR transitional periods. 6 10 11 As a secondary objective, we sought to further examine the effect of prior EHR experience on usability perspectives, which elsewhere has been suggested as a limited predictor of future productivity and usability patterns. 12 This research study can provide valuable insight to inform EHR optimization initiatives inclusive of different staff usability perceptions and needs.

## Methods

### Study Design and Setting

We conducted a cross-sectional study of ambulatory patient-facing health professionals at NYP-affiliated CU and WC campuses completing a customized usability survey that had been administered across multiple settings prior to each campus' EpicCare implementation. In February 2020, CU inpatient and ambulatory settings initiated the transition of Allscripts Sunrise, Allscripts Touchworks, and other homegrown EHRs to EpicCare EHR. The CU preimplementation survey was administered over a 5-week period prior to CU EpicCare implementation at ambulatory and inpatient settings between October 2019 and December 2019. In October 2020, the WC inpatient setting made a similar transition from Allscripts Sunrise EHR, however, the WC ambulatory practices had already implemented EpicCare EHR in 2001 and continued to use this EHR. 10 The WC preimplementation survey was administered over a 5-week period prior to WC EpicCare implementation at inpatient settings between October 2020 and November 2020. OPs were defined as physicians, physician assistants, and nurse practitioners. Questionnaires with less than 90% survey item completion were excluded from analysis and all responses were self-reported.

### Survey Instrument

We adapted our survey instrument from a 19-question electronic survey ( Supplementary Fig S1 ) utilizing the Health-ITUES constructs QWL, PU, PEU, and UC and added the CSSA construct to provide information about user perceptions about common situations presented in the EHR. Responses were scored based on Likert scale ratings ranging from 1 (strongly disagree) to 5 (strongly agree) with higher scores denoting higher usability. Demographic information was also collected including clinical role, specialty, setting, years of experience, and prior EHR use. Respondents were able to select either inpatient, ambulatory, emergency department, or a combination thereof as a practice setting; however, only subjects exclusively reporting ambulatory setting were included for analysis.

### Analysis

One-way and two-way analysis of variance tests and pairwise comparisons with Bonferroni correction within groups were conducted on responses based on demographic information. All study aspects were approved by both CU and WC Institutional Review Boards.

## Results

### Cohort Demographics

Of 11,887 CU clinical staff surveyed, 3,598 respondents (30%) completed at least 90% of survey items, and of these 1,666 identified as solely ambulatory. Of 10,810 WC clinical staff surveyed, 2,754 respondents (25%) completed at least 90% of survey items, and of these 1,065 identified as solely ambulatory. A total of 1,666 and 1,065 ambulatory CU and WC respondents, respectively, were included for study. The total number of eligible ambulatory providers could not be reliably ascertained at the time of study as survey respondents self-identified and the initial survey distribution list did not otherwise specify job type. Select demographic statistics such as age and gender were similar between CU and WC groups, while OP role type, professional degree, higher years of EHR experience, and certain specialties such as anesthesiology, medicine, radiology, and surgery were slightly more prevalent among WC than CU respondents ( Table 1 ).

**Table 1:** CU and WC survey respondent demographic information

	CU ( *n* = 1,666)	WC ( *n* = 1,065)	*p* -Value a
Ordering providers	777 (47%)	546 (51%)	< 0.001
Nonordering providers	867 (52%)	447 (42%)	< 0.001
Age between 25 and 64	1,410 (85%)	908 (85%)	0.4
Gender male/female	441 (27%)/1,153 (70%)	314 (30%)/697 (66%)	0.2
Highest degree			0.002
Associate's degree	162 (10%)	63 (6%)	
Bachelor's degree	333 (20%)	214 (21%)	
Professional degree	566 (34%)	415 (40%)	
Specialty b			
Anesthesiology	44 (3%)	55 (6%)	
Dermatology	18 (1%)	7 (1%)	
Medicine	268 (17%)	195 (19%)	
Neurology	51 (3%)	26 (3%)	
OB/GYN	79 (5%)	45 (5%)	
Ophthalmology	38 (2%)	27 (3%)	
Pediatrics	193 (12%)	73 (7%)	
Psychiatry	96 (6%)	73 (7%)	
Radiology	111 (7%)	113 (11%)	
Rehabilitation Medicine	50 (3%)	5 (1%)	
Surgery c	167 (10%)	123 (12%)	
Years of clinical experience			0.02
Less than 1 year	51 (3%)	19 (2%)	
1–10 years	634 (38%)	419 (39%)	
11 years or more	946 (57%)	599 (56%)	
Years with current EHR			0.011
Less than 1 year	192 (12%)	136 (13%)	
1–10 years	1,136 (68%)	677 (64%)	
11 years or more	281 (17%)	212 (20%)	

Abbreviations: OB/GYN, obstetrics/gynecology; CU, Columbia University College of Physicians and Surgeons; EHR, electronic health record; WC, Weill Cornell Medical College.

a Pearson's chi-squared test.

b Not shown include additional specialties collectively < 5% of entire sample such as dentistry, laboratory, nutrition, occupational therapy, speech and language pathology, physical therapy, and social work.

c Includes general, colorectal, neurosurgery, plastic surgery, otorhinolaryngology, orthopaedic, urology, and vascular.

### Patterns of Likert Scale Responses

Likert scale survey responses for all ambulatory staff stratified by campus demonstrated significantly higher usability at WC than CU across all constructs ( Fig. 1 , *p* -values range from <0.001 to 0.003). The PU and UC constructs demonstrated the greatest variation between campuses ( *β* = 0.47, 0.44, respectively). The CSSA construct demonstrated the least variation between campuses ( *β* = 0.12).

**Fig. 2:**
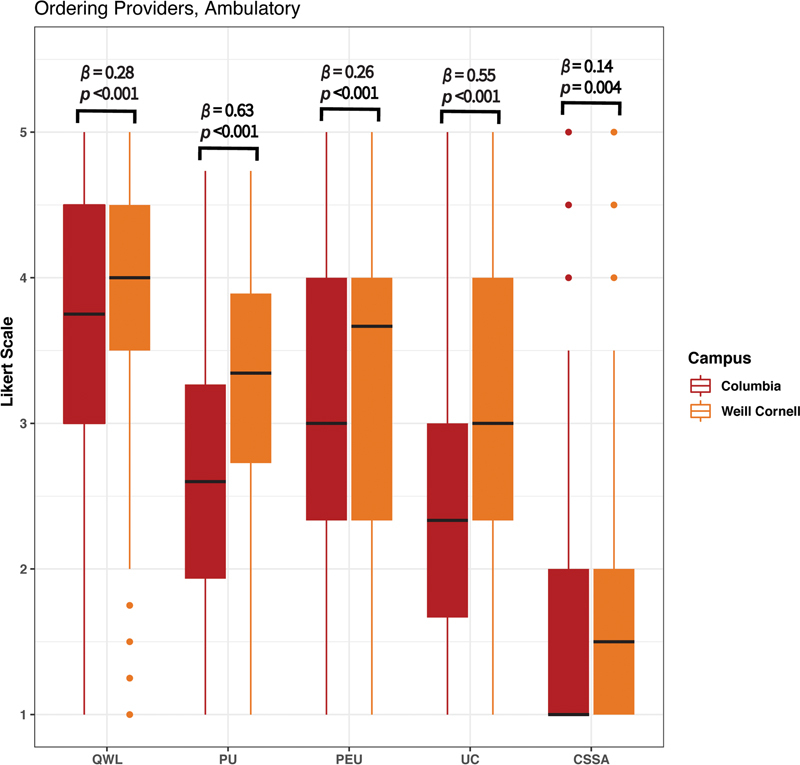
Ambulatory responses of ordering providers (OPs) at both Columbia University College of Physicians and Surgeons (CU) and Weill Cornell Medical College (WC).

**Fig. 3:**
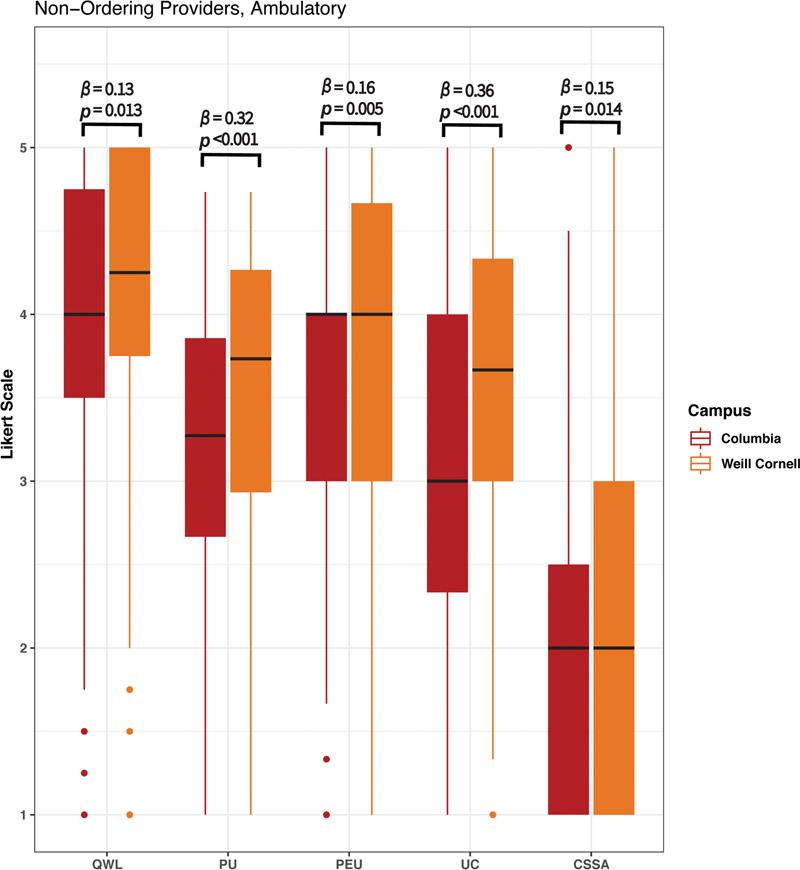
Ambulatory responses of non-ordering providers (OPs) at both Columbia University College of Physicians and Surgeons (CU) and Weill Cornell Medical College (WC).

Ambulatory clinical staff were substratified by provider role. Survey responses for OPs and non-OPs stratified by campus likewise demonstrated significantly higher usability at WC than CU across all constructs ( Figs. 2 and 3 , *p* -values range from < 0.001 to 0.014). Among both OPs and non-OPs, the PU and UC constructs demonstrated the greatest variation between campuses ( *β* = 0.63 and 0.55, respectively, among OPs, and *β* = 0.32 and 0.36, respectively, among non-OPs). Among OPs, the CSSA construct demonstrated the least variation between campuses ( *β* = 0.14). Among non-OPs, the QWL and CSSA constructs demonstrated the least variation between campuses ( *β* = 0.13 and 0.15, respectively). Variation in usability constructs between campuses was most pronounced for OPs.

### Prior Electronic Health Record Experience

Comparison of categorical survey responses among all ambulatory respondents to years of prior EHR experience demonstrated limited significant associations with construct usability perceptions ( Table 2 ). Additional years of prior EHR use was significantly associated with overall increasing perceived usability among the PU, PEU, and UC constructs, but was persistently negative for the CSSA construct. There were no further significant or consistent associations for the remaining constructs among OPs or non-OPs subgroups at both CU and WC.

**Table 2:** Select associations of prior EHR use and usability constructs among all ambulatory respondents

	All ambulatory respondents ( *n* = 2,731)			
	Prior EHR experience (y)	*β*	95% CI	*p* -Value
QWL	11–20	0.2	0.07, 0.33	0.002
PU	1–2	–0.21	–0.35, –0.07	0.003
	3–5	–0.13	–0.25, 0.00	0.049
	11–20	0.20	0.06, 0.34	0.004
	21 or more	0.37	0.09, 0.65	0.008
PEU	3–5	0.17	0.02, 0.31	0.024
	6–10	0.19	0.04, 0.33	0.01
	11–20	0.30	0.14, 0.45	<0.001
	21 or more	0.46	0.14, 0.77	0.005
UC	1–2	–0.29	–0.45, –0.13	<0.001
	3–5	–0.27	–0.41, –0.12	<0.001
	6–10	–0.34	–0.48, –0.20	<0.001
	21 or more	0.37	0.05, 0.68	0.024
CSSA	1–2	–0.27	–0.41, –0.13	<0.001
	3–5	–0.32	–0.44, –0.19	<0.001
	6–10	–0.25	–0.37, –0.12	<0.001
	11–20	–0.23	–0.37, –0.10	<0.001

Abbreviations: CI, confidence interval; CSSA, Cognitive Support and Situational Awareness; EHR, electronic health record; PEU, Perceived Ease of Use; PU, Perceived Usefulness; QWL, Quality of Work Life; UC, User Control.

## Discussion

This study sought to characterize usability perceptions among ambulatory clinical staff utilizing EpicCare and various iterations of Allscripts at a major academic health care system prior to EHR transition. We found that OPs consistently denoted less usability overall, but were more affected by EHR systems than non-OPs, and that prior EHR experience did not reliably predict usability perceptions.

To date, there have been many patient care and quality improvement efforts premised on the improvement of EHR workflows, accessibility, and usability. 13 Despite the benefits of such initiatives in aspects of clinical data retrieval, storage, and cost-saving, EHRs and clinical decision support tools are often perceived as lagging in their potential to optimally leverage complementary and contemporary technologies in a complex and rapidly changing clinical work environment. 13 14 15 As central interfaces requiring user interaction for nearly all aspects of clinical care, EHRs can either greatly contribute to downstream successes or inadvertently potentially interfere with desired end goals simply based on their design. 13 16 One study of pediatric EHRs found that as many as two-thirds of safety reports were related to usability issues with the EHR. 17 A further complication to optimizing EHRs inevitably arises from the fact that different users require and expect different functions based on their role, setting, and specialty within the health care system.

As expected from prior study data by the same team, our study showed significant differences in EHR usability perceptions based on clinical role and EHR system. Although OPs denoted less usability compared to non-OPs, OP usability was more affected by the EHR system(s) used as evidenced by CU and WC result stratification. The PU and UC constructs accounted for the largest differences in usability perceptions among OPs using either EHR system. These results suggest a possible greater perceived usability for EpicCare to perform tasks that are often more specific to OPs in the ambulatory setting, such as coordinating care, note documentation, information review, and error correction or prevention.

The CSSA construct accounted for the lowest Likert scale rankings for most users of both campuses. These results suggest that among all providers of either EHR system there were persistently low evaluations for ease of navigating multiple EHR tabs, user interface, and cognitive burden reduction. These constructs are particularly relevant to adverse EHR outcomes identified in literature related to clinical burnout, job dissatisfaction, and adverse patient safety events. 1 2 3 18

In our study, prior clinical and EHR experience differed slightly between CU and WC groups overall, but this seems unlikely to have been responsible for the differences in usability attributed to EHR systems as these group differences were small (i.e., ≤ 4%) and somewhat inconsistent (i.e., larger representation of “less than 1 year” and “11 years or more” simultaneously for both categories). Furthermore, dedicated analysis among all ambulatory respondents demonstrated limited associations with construct usability perceptions related to care coordination, note documentation, information review, EHR learnability, and error correction or prevention as there were no significant or consistent associations identified for either OPs or non-OPs at CU or WC. Although expected to influence usability perceptions in some manner, it is possible then that prior EHR use may be less impactful on perceptions than the effect of provider role or EHR system.

Unlike our study, which differentiated among five usability constructs across two separate EHR systems, a recent study of usability associations among nursing staff in over 300 hospitals demonstrated persistent associations between suboptimal EHR usability, staff burnout, and adverse outcomes despite averaging usability Likert scores, suggesting any aspect of negative usability can potentially offset unique advantages of different EHR designs. 18 Perhaps reflective of this implication, another study surveying clinician attitudes has demonstrated stagnant or even decreasing satisfaction with EHR usability metrics across time despite vendor improvements and updates. 19

In conclusion, potential optimism regarding the favorability of certain EHR systems on select aspects of usability perceptions must be tempered by persistent user-identified concerns related to critical cognitive constructs regardless of prior experience. Our study suggests these constructs may be most responsible for suboptimal EHR usability overall, although future work should further explore ways to address cognitive burden impeding user workflows. There were significant differences in EHR usability perceptions based on clinical role and EHR system, particularly for OPs coordinating care and engaging in note documentation, information review, or error correction. Our results support the findings of other studies that have called for EHR implementation strategies recognizing such distinctions as well as policies that promote good faith efforts to report usability and safety concerns in this area. 20 21

There were several limitations to this study. A major limitation is that survey respondents did not explicitly specify either EpicCare or Allscripts in their survey responses; however, we believe restricting respondents to the ambulatory setting most likely controlled for the EHR system evaluated as the available EHRs for each setting were known a priori. Furthermore, although specialty distribution was generally similar in both campus groups, differences in specialty make-up could also entail specialty specific tasks and workflows that influence usability perceptions in unique ways. Such specialty specific differences were beyond the scope of our study and unlikely to affect study outcomes given limited differences in specialty make-up, but could warrant future research. Overall survey response rate was low, potentially reflecting nonresponse bias, and it is unclear to what degree our results may misrepresent other patterns of responses among ambulatory staff that were unable to be included. Alternatively, our study may in fact reflect salient differences in usability perceptions while also adequately accounting for a central tendency bias of our population. Although many usability differences were shown to be significant, the overall effect size was small, perhaps in part due to the frequency of Likert scale “3” scoring or indifferent survey responses. Finally, unlike CU, the WC preimplementation survey occurred during the coronavirus disease 2019 pandemic which may have influenced usability perception responses among staff facing unique workflow challenges.

Future work will include postimplementation survey results at CU and WC for further characterization of evolving usability perceptions. While our study focused on only the ambulatory setting of care, it seems likely that other areas of care will also be affected. Our results here may be predictive of perceived successes and shortcomings of the final EHR transition and guide appropriate interventions. Such studies are necessary given the relative lack of literature on EHR-to-EHR transitions 22 at a time when an “emerging EHR monoculture” 23 seems likely to play an ever larger role for health care organizations.

## Conclusion

While EHRs have been associated with many improvements, a growing body of literature has also linked EHRs to several adverse outcomes due to usability. This study characterized usability perceptions of over 2,700 clinical staff utilizing EpicCare and various iterations of Allscripts in the ambulatory settings of a major academic health care system prior to EHR transition. OPs consistently denoted less usability overall, but appear more affected by differing EHR systems than non-OPs. While there was greater perceived usability for EpicCare to perform tasks related to care coordination, documentation, and error prevention, there were persistent shortcomings identified regarding tab navigation and cognitive burden reduction which may have implications on patient care. Prior EHR experience did not significantly affect primary study results. Assessing differences in usability perceptions may provide insight into customized strategies for improving EHR usability for clinical staff with different roles. Future work will include postimplementation survey results for further characterization of evolving usability perceptions.

## Clinical Relevance Statement

Usability perceptions of EHRs can be affected by user roles and EHR system. In our study, OPs consistently denoted less usability overall and were more affected by EHR system than non-OPs. Despite apparent usability differences related to EHR system, we identified persistent shortcomings regarding cognitive burden reduction which have implications for clinical burnout, job dissatisfaction, and patient adverse events.

## Multiple-Choice Questions

1. Which of the following constructs was evaluated most negatively by users?
  - Quality of Work Life
  - Perceived Ease of Use
  - Cognitive Support and Situational Awareness
  - User Control
  **Correct Answer:** The correct answer is option c.
  Explanation: the Cognitive Support and Situational Awareness construct accounted for not only the lowest Likert scale rankings for most users, but also a smaller difference between user role types. This construct reflects the ease of navigating multiple EHR tabs, user interface, and cognitive burden reduction, which has been associated in literature to clinical burnout, job dissatisfaction, and adverse patient safety events.
2. Which of the following users' usability perceptions appeared to be most impacted by EHR system?
  - Ordering providers
  - Nonordering providers
  - Users with many years of experience
  - Users with fewer years of experience
  **Correct Answer:** The correct answer is option a.
  Explanation: in our study, ordering providers (physicians, physician assistants, and nurse practitioners) consistently denoted less usability overall and were more affected by EHR system than nonordering providers. Prior EHR experience among each category of ambulatory respondents demonstrated no significant or consistent associations with differences in usability perceptions.
