# Supplementary material for: Assessing Usability and Ambulatory Clinical Staff Satisfaction with Two Electronic Health Records
Source: Appl Clin Inform. 2023 Jun 28;14(3):494–502. doi: 10.1055/a-2074-1665 (PMC10306987; doi:10.1055/a-2074-1665)
Supplement: Supplementary file 1 — Supplementary Material [file 10-1055-a-2074-1665-s202301ra0003.pdf]

	Item	Concept
**Quality of Work Life**		
1.	The current EHR has been a positive addition to providing clinical care **within my field**.	System impact-career mission
2.	The current EHR has been a positive addition to our **organization's ability** to support continuity of care.	System impact-organizational level
3.	a. The current EHR is an important part of my **clinical documentation** process.<br>b. The current EHR is an important part of my **patient information review** process.	System impact-personal level
**Perceived Usefulness**		
4.	The current EHR is useful for supporting my ability to **coordinate care** across multiple specialties within my organization.	General usefulness
5.	The current EHR presents a supportive environment for interdisciplinary collaboration **on clinical documentation.**	General usefulness
6.	The current EHR **supports the review of information** provided by other disciplines and specialties.	General usefulness
7.	a. I am satisfied with the current EHR for **clinical documentation**.<br>b. I am satisfied with the current EHR for **patient information review**.	General satisfaction
8.	Have you used other clinical documentation systems in the past (e.g., other EHRs or paper documentation)?	Logic question
9.	a. Compared to other documentation systems (e.g., other EHRs or paper documentation) I have used, using the current EHR makes it easier to effectively **document** patient information.<br>b. Compared to other documentation systems (e.g., other EHRs or paper documentation) I have used, using the current EHR makes it easier to effectively **review** patient information.<br>c. Compared to other documentation systems I have used (e.g., other EHRs or paper documentation), using the current EHR enables me to **document** patient information more quickly.<br>d. Compared to other documentation systems I have used (e.g., other EHRs or paper documentation), using the current EHR enables me to **review** patient information more quickly.	Productiveness
10.	I perform **clinical documentation** tasks quickly because of the current EHR.	Performance speed
11.	I perform **patient information review** tasks quickly because of the current EHR.	Performance speed
12.	Using the current EHR increases continuity of care between my organization and others.	Productiveness
13.	a. The current EHR allows me to access all necessary data for **my clinical documentation.**<br>b. The current EHR allows me to access all necessary data for **adequate patient information review**.	Information needs
**Perceived Ease of Use**		
14.	The current EHR was easy to **learn.**	Learnability
15.	The current EHR was easy to **use.**	Competency
16.	It was easy for me to become proficient using the current EHR.	Ease of use
**User Control**		
17.	The current EHR provides helpful on-screen messages and alerts to prevent clinical errors.	Error prevention
18.	Whenever I make an error using the current EHR, I can fix the mistake easily and quickly.	Error prevention
19.	Information such as on-line help, on-screen messages and alerts provided with the current EHR is clear.	Information needs
**Cognitive Support and Situational Awareness**		
20.	I need to navigate multiple tabs and pages in the EHR to perform clinical documentation about my patients.	Cognitive support and situational awareness
21.	I need to navigate multiple tabs and pages in the EHR for information that will give me a complete picture of my patient.	Cognitive support and situational awareness

Supplementary Fig. S1 Survey instrument.

 © 2023. The Author(s).
